# Editorial: Physiological and biomechanical determinants of swimming performance—volume 2

**DOI:** 10.3389/fspor.2023.1142336

**Published:** 2023-02-09

**Authors:** Flávio De Souza Castro, Pedro Figueiredo, Argyris G. Toubekis, Tiago M. Barbosa, Carla McCabe

**Affiliations:** ^1^ School of Physical Education, Physioterapy and Dance, Universidade Federal do Rio Grande do Sul, Porto Alegre, Brazil; ^2^ Department of Physical Education, College of Education, United Arab Emirates University, Al Ain, Abu Dhabi, United Arab Emirates; ^3^ National and Kapodistrian University of Athens, Athens, Greece; ^4^ Department of Sport Science, Instituto Politécnico de Bragança, Bragança, Portugal; ^5^ Department of Human and Health, Research Centre in Sports Sciences, Covilhã, Portugal; ^6^ School of Sport, Ulster University, Belfast, United Kingdom

**Keywords:** assessment, performance, sports, training, develoment

**Editorial on the Research Topic** Physiological and biomechanical determinants of swimming performance—volume 2

The objective of this Research Topic was to develop and strengthen evidence of training and swimming performance to increase scientific knowledge in the area, considering that understanding the biomechanical, physiological, and neuromuscular determinants of swimming performance is still challenging. This way, 13 manuscripts have been reviewed and approved for this research topic (volume II). We can categorize the 13 manuscripts into three major areas of swimming research: physiology and prescription; biomechanics; performance assessment and prediction. Furthermore, we highlight that 10 of the manuscripts were carried out with the participation of at least two research institutions, often from different countries, which may demonstrate the need for international interchange and exchange of ideas and methodologies across researchers and laboratories.

Concerning physiological aspects of swimming training and performance, studies focused on oxygen uptake kinetics, back-extrapolation reliability, and critical speed. Almeida et al. (*Time Limit and VO_2_ Kinetics at Maximal Aerobic Velocity: Continuous vs. Intermittent Swimming Trials*) assessed 22 male swimmers in an incremental protocol to estimate, among others, the maximal aerobic velocity, then applied intermittent and continuous swimming protocols, both at maximal aerobic velocity. They noted that: (i) intermittent trials training is preferable rather than continuous training for aerobic capacity; and (ii) $\dot{V}O_{2}$ kinetics do not appear to influence time spent at severe intensity domain in both intermittent and continuous swimming training. However, the $\dot{V}O_{2}$ and its kinetic measurements in swimming still generate debate about their methods. Thus Massini et al. (*The reliability of back-extrapolation in estimating* $\dot{V}O_{2}$*peak in different swimming performances at severe intensity domain*) estimated $\dot{V}O_{2}$ by back-extrapolation for 20 swimmers after (i) an incremental intermittent step protocol and (ii) a 200 m single-trial. Among the results, they found that the initial phase of the $\dot{V}O_{2}$ recovery profile provided different (although reliable) conditions to the estimate of $\dot{V}O_{2}$peak with back-extrapolation procedures, which accounted for the effect of anaerobic release on $\dot{V}O_{2}$ off-kinetics, but compromised, exceptionally, the $\dot{V}O_{2}$peak estimate in 200 m single-trial. Focusing on training prescription with critical speed, Raimundo et al. (*Modeling the Expenditure and Reconstitution of Distance Above Critical Speed During Two Swimming Interval Training Sessions*) suggest that the time constant of the reconstitution of the maximum distance that can be performed above critical speed is not constant during two high-intensity interval sessions with the same recovery intensity.

The studies that focused on the biomechanics of swimming, in this special volume, can be grouped into three main topics: (i) inertial and pressure systems; (ii) tumble turn; breaststroke pullout; and undulatory underwater speed; and (iii) active drag, propulsion and kinematics. Rad et al. (*Monitoring weekly progress of front crawl swimmers using IMU-based performance evaluation goal metrics*) investigated inertial measurement unit (IMU) with a single IMU on the 16 swimmers' sacrum during training sessions, specifically along ten weeks in 25-m all-out front crawl. Five goal metrics from the IMU signals representing the swimmer's performance in the swimming phases (wall push-off, glide, stroke preparation, free-swimming) and in the entire lap were estimated. The results showed that the goal metrics for the free-swimming phase and the entire lap predicted the swimmer's progress well. Regarding pressor sensors, Santos et al. (*Reliability of using a pressure sensor system to measure in-water force in young competitive swimmers*) analyzed the front crawl over 25-m all-out of 15 age-group swimmers with the pressure sensor system (Aquanex System). They concluded that the system seems to be a reliable device for measuring the hand resultant force during front crawl in young swimmers and can be used to monitor the changes over time.

Concerning the tumble turn performance, Koster et al. *(Implications of the choice of distance-based measures in assessing and investigating tumble turn performance*) intended to understand better the implications of choosing a particular distance-based performance measure for assessing and investigating tumble turn performance in freestyle swimming. In this way, 2,813 turns performed by 160 swimmers were analyzed. The results revealed that performance measures with short(er) distances are more sensitive to changes in the adaptation time and reflect the wall contact time better than performance measures with long(er) distances, which in contrast, are more useful if the focus is on the approach speed prior to the turn. David et al. (*Improving tumble turn performance in swimming—the impact of wall contact time and tuck index*) examined the effect of wall contact time and tuck Index on tumble turn performance and their interrelations by experimentally manipulating both variables. The results underscored the importance of wall contact time and tuck Index of the tumble turn performance, as well as their interrelations with other performance determining variables in this regard, with the importance of individual tuning. Regarding the breaststroke pull-out, McCabe et al. (*The Characteristics of the Breaststroke Pullout in Elite Swimming)* characterized the underwater breaststroke pullout technique trends and assessed the effectiveness of each technique as utilized by elite male and female swimmers. The study found no difference in performance outcome for each pullout technique, indicating that one's individual preference should guide technique selection. Concerning the undulatory underwater movement, Kuhn and Legerlotz (*Ankle joint flexibility affects undulatory underwater swimming speed*) investigated the impact of ankle joint flexibility on swimming velocity and kick efficiency during undulatory underwater by comparing kinematics of swimming trials with reduced, normal, and enhanced maximum angles of plantar flexion. Swimming velocity and kick efficiency did not differ between normal and increased plantar flexion. The results suggest that undulatory underwater velocity is affected by impaired plantar flexion.

Concerning active drag, Lopes et al. (*Numerical and experimental methods used to evaluate active drag in swimming: A systematic narrative review*) performed a systematic review to update the body of knowledge on active drag in swimming through numerical and experimental methods. Seventy-five studies on active drag in swimming and the methodologies applied to study them were analyzed and kept for synthesis. There were significantly fewer numerical studies than experimental ones. Based on the complexity of active drag, studying this phenomenon must continue to improve swimming performance. About the propulsion, Morais et al. (*Understanding the role of propulsion in the prediction of front-crawl swimming velocity and in the relationship between stroke frequency and stroke length*) aimed to: (i) determine swimming velocity based on a set of anthropometric, kinematic, and kinetic variables, and; (ii) understand the stroke frequency–stroke length combinations associated with swimming velocity and propulsion in young sprint swimmers. Swimming velocity was predicted by an interaction of anthropometrics, kinematics, and kinetics. Faster velocities in young sprinters of both sexes were achieved by an optimal combination of stroke frequency–stroke length. The propulsion data showed the same trend. The highest propulsion was not necessarily associated with higher velocity achievement.

Regarding the performance assessment and prediction, considering the 400-m front crawl test as a useful tool to assess aerobic power and capacity, Correia et al. (*Kinematic, arm-stroke efficiency, coordination, and energetic parameters of the 400-m front-crawl test: a meta-analysis*) provided a meta-analysis assessing representative variables for the kinematic, arm-stroke efficiency, coordination, and energetic parameters of the 400-m front crawl test. High heterogeneity (>75%) was found among the outcome parameters in the studies on the meta-analysis. The average speeds seem to be the most responsible and influential in the arm-stroke efficiency, coordination, and energetic parameters for improved 400-m front-crawl performance. Finally, Born et al. (*Performance development of European swimmers across the Olympic cycle*) quantified the performance development of race time and key performance indicators of European swimmers across the last Olympic cycle (from 2016 to 2021) and provided reference values for long-course swimming pool events for both sexes from 50 m to 1,500 m including butterfly, backstroke, breaststroke, freestyle, and individual medley. Individual events from the 2016 and 2021 European swimming championships were included in the analysis. Among the results, clean swimming velocities were faster in 12 (males) and 5 (females) events. For alternating swimming strokes, i.e., backstroke and freestyle, effect sizes indicated improved swimming efficiency with an inverse relationship between reduced stroke rate and increased distance per stroke.

In summary, this issue advances the knowledge in topics of practical importance related to swimming performance. It highlights the contribution of several research areas, including physiology, biomechanics, new technologies and race analysis and, performance progression. In terms of physiology, training planning of continuous or intermittent sessions, oxygen kinetics, and critical speed remain hot topics in swimming research. In biomechanics, analyzing specific parts and components of a race remains critical and will help a substantial performance improvement. New technologies using reliable devices will help improve training quality, and we expect further improvement soon.

## Author contributions

FSC, PF, AT, TB, and CM wrote and proofread this editorial. All authors contributed to the article and approved the submitted version.

## Conflict of interest

The authors declare that the research was conducted in the absence of any commercial or financial relationships that could be construed as a potential conflict of interest.

## Publisher's note

All claims expressed in this article are solely those of the authors and do not necessarily represent those of their affiliated organizations, or those of the publisher, the editors and the reviewers. Any product that may be evaluated in this article, or claim that may be made by its manufacturer, is not guaranteed or endorsed by the publisher.

